# Factors associated with school absenteeism in adolescents with mental illness: a multivariate analysis

**DOI:** 10.3389/fpsyt.2026.1888051

**Published:** 2026-09-25

**Authors:** Xiaofei Zhou, Dan Liu, Huiyi Wang, Bo Li, Jiewei Zhang, Ying Wang

**Affiliations:** 1 Department of Psychosomatic Medicine, Honghui Hospital, Xi’an Jiaotong University, Xi’an, Shaanxi, China; 2 Department of Psychosomatic Medicine, Xi'an International Medical Center Hospital, Xi’an, Shaanxi, China

**Keywords:** adolescent mental illness, prognosis, retrospective cohort study, risk factors, school absenteeism, social support

## Abstract

**Background:**

school absenteeism is a common functional impairment among adolescents with mental disorders, yet its associated factors remain limited. This study aimed to identify key demographic, clinical, and social factors associated with school absenteeism in this population.

**Methods:**

A retrospective cohort study was conducted among 217 adolescents (12–18 years) with mental illness, categorized into school-attending (n=163) and school-suspended (n=54) groups. Baseline data on demographics, clinical characteristics, social support, and family factors were collected. Univariate analyses were used to compare group differences, followed by multivariate logistic regression and restricted cubic spline (RCS) analysis to identify independent predictors of school absenteeism.

**Results:**

In univariate analysis, school-suspended adolescents had significantly lower social support scores (36.2 ± 10.9 vs. 45.5 ± 12.2, p<0.001), higher rates of parental divorce (46.3% vs. 22.1%, p<0.001), and a higher proportion of pre-hospitalization school absenteeism (51.9% vs. 31.3%, p=0.006). Multivariate logistic regression revealed that parental divorce (OR = 3.04, 95%CI=1.59–5.83, p=0.001), pre-hospitalization school absenteeism (OR = 2.37, 95%CI=1.26–4.43, p=0.007), and longer disease course (OR = 1.02, 95%CI=1.00–1.04, p=0.022) were independent risk factors for school absenteeism. Conversely, higher social support was a strong protective factor: compared to the low social support group (score 17–34), the medium (35–51) and high (52–85) support groups had markedly lower odds of school absenteeism (OR = 0.24, 95%CI=0.08–0.74, p=0.013; OR = 0.05, 95%CI=0.01–0.24, p<0.001, respectively). RCS analysis confirmed a linear negative association (P for non-linearity=0.189), with higher social support scores correlating with progressively lower suspension risk.

**Conclusions:**

Parental divorce, pre-hospitalization school absenteeism, and longer disease course are independent risk factors for school absenteeism in adolescents with mental illness, while higher social support acts as a robust protective factor. These findings highlight the need for targeted interventions addressing family stability, social support enhancement, and early intervention for pre-existing school impairment to reduce school absenteeism and promote functional recovery in this vulnerable population.

**Clinical trial registration:**

https://clinicaltrials.gov/, identifier ChiCTR2100046396.

## Introduction

Adolescence is a critical developmental period marked by profound biological, psychological, and social changes, during which mental health disorders often emerge with high prevalence and functional consequences. The younger onset of mental disorders has become a pressing issue in the global public health domain (1, 2). Studies indicate a significant increase in the incidence of mental disorders such as depression (3), anxiety, and bipolar disorder (4) among adolescents and young adults. According to World Health Organization (WHO) estimates, approximately one in six children and adolescents worldwide are afflicted by mental health issues, with almost half of instances beginning before age 20, the average global coverage of 10-19-year-old mental disorder incidence data was 14% (5, 6). school absenteeism represents a severe and persistent functional impairment among adolescents with mental illness, disrupting educational attainment, social development, and long-term life outcomes.

In the prognosis evaluation and rehabilitation management of adolescents with mental illness, school re-entry rate and academic functional recovery are among the core outcome measures jointly concerned by clinicians and families. Parents universally hold the perception that school attendance is of great significance, firmly believing that continuous school education is the key to ensuring their children’s future social integration, career development, and self-actualization. This perception directly influences the family’s engagement in rehabilitation treatment, decision-making regarding their child’s academic arrangement, and adherence to therapeutic protocols, thereby emerging as a crucial psychosocial factor and environmental variable impacting the prognosis of adolescents with mental illness (7). Previous epidemiological data indicate that up to one-quarter of hospitalized adolescent psychiatric patients experience school absenteeism, yet the modifiable and non-modifiable factors driving this outcome remain incompletely understood.

The occurrence of adolescent mental health problems is the result of the synergistic effect of multiple factors (8) A single risk factor cannot fully explain its pathogenesis. Therefore, it is necessary to integrate multi-level risk factors, including individual, family, school, and social factors, for systematic analysis to accurately reveal the essence and internal correlation of the problem. Meanwhile, the rehabilitation process of adolescents with mental illness is also inseparable from the collaborative support of multiple parties. It requires families, schools, and society to form a joint force and build a comprehensive rehabilitation support system to provide guarantee for the patients’ rehabilitation and social reintegration.

Family structure, particularly parental marital status, has been recognized as a key contextual factor shaping adolescent well-being (9). Parental divorce is associated with increased psychological distress, reduced family cohesion, and heightened risk of academic disengagement (10, 11).

, but its specific contribution to school absenteeism in mentally ill adolescents has not been fully quantified in multivariate models. Additionally, pre-hospitalization school functioning and disease-related factors, such as illness duration and diagnostic subtype, may create a cumulative burden that perpetuates school absenteeism, though their independent effects require adjustment for potential confounding variables (12).

Social support, a multifaceted construct encompassing emotional, instrumental, and informational resources from family, peers, and school, is widely recognized as a protective factor against adverse mental health outcomes. While prior studies have linked higher social support to improved treatment adherence and reduced symptom severity (13, 14), its role in mitigating school absenteeism risk among adolescents with mental illness remains understudied, especially in terms of independent effects after accounting for family and clinical covariates.

Most prior studies have focused on correlations between parental divorce, social support, adolescent mental health outcomes, and non-suicidal self-injury (NSSI) with few integrating adverse school outcomes (i.e., school absenteeism) into a unified analytical framework, particularly in clinical adolescent samples (15, 16). Furthermore, the mechanisms and interactions among these variables remain insufficiently investigated (17, 18). To bridge this gap, the present study conducted a comprehensive multivariate analysis of factors associated with school absenteeism in a cohort of hospitalized adolescents with mental illness. Specifically, we sought to answer three core research questions: What demographic, clinical, and family factors independently predict school absenteeism? Is social support independently associated with a reduced risk of school absenteeism after adjusting for covariates? Does the relationship between social support and school absenteeism follow a linear dose-response pattern?

The novelty of this study is threefold. First, we utilized a specific sample of Chinese hospitalized adolescents with detailed clinical profiles, a population understudied in this context. Second, we incorporated multidimensional outcome indicators, focusing on practical functional recovery (return-to-school) as the key endpoint. Third, our findings offer direct clinical implications, providing empirical evidence to guide targeted interventions for maintaining family stability, enhancing social support, and delivering early intervention for at-risk youth, ultimately promoting functional recovery in this vulnerable population.

## Methods

### Study design and population

A retrospective cohort study was conducted among 217 hospitalized adolescents (12–18 years) with mental illness. The available population comprised adolescent patients diagnosed with emotional and behavioral disorders who were admitted to the Department of psychosomatic medicine at Xi’an International Medical Center Hospital between July 2019 and December 2022. Patients were enrolled according to strict inclusion and exclusion criteria. Inclusion criteria were: (1) aged 12–18 years; (2) diagnosed with a mental disorder according to ICD-10 criteria; and (3) available complete medical records. Exclusion criteria were: (1) hospitalization duration < 24 hours; and (2) loss to follow-up. A total of 223 medical records were initially screened. After excluding 2 cases with hospitalization < 24 hours and 4 cases lost to follow-up, 217 patients were finally enrolled, yielding an enrollment rate of 97.31%. Extracted data included age, sex, only-child status, educational level, parental marital status, Adverse Childhood Experiences (ACEs) history, non-suicidal self-injury (NSSI) history, suicidal ideation and attempts, family history of mental and somatic illness (19), psychiatric diagnosis, age of onset, disease course, pre-hospitalization school absenteeism status, and social support scores. The final follow-up visit occurred one month after discharge. Return-to-school status was evaluated via physician-led telephone interviews and outpatient re-examinations. Adolescents who had returned to school were classified as having a good prognosis, whereas those who remained on school absenteeism were classified as having a poor prognosis. Regarding the 4 lost-to-follow-up cases, 2 could not be contacted due to changed contact information after discharge, and 2 refused to participate in the follow-up assessment for personal family reasons (**Figure 1**). No significant differences in baseline demographic or clinical characteristics were found between the lost-to-follow-up and included participants, indicating no substantial impact on the overall results. Ethical approval for this study was granted by the Medical Ethics Committee of Xi’an International Medical Center Hospital (approval No.2021009). The retrospective study was registered with the Chinese Clinical Trial Registry (registration No. ChiCTR2100046396).

**Figure 1:**
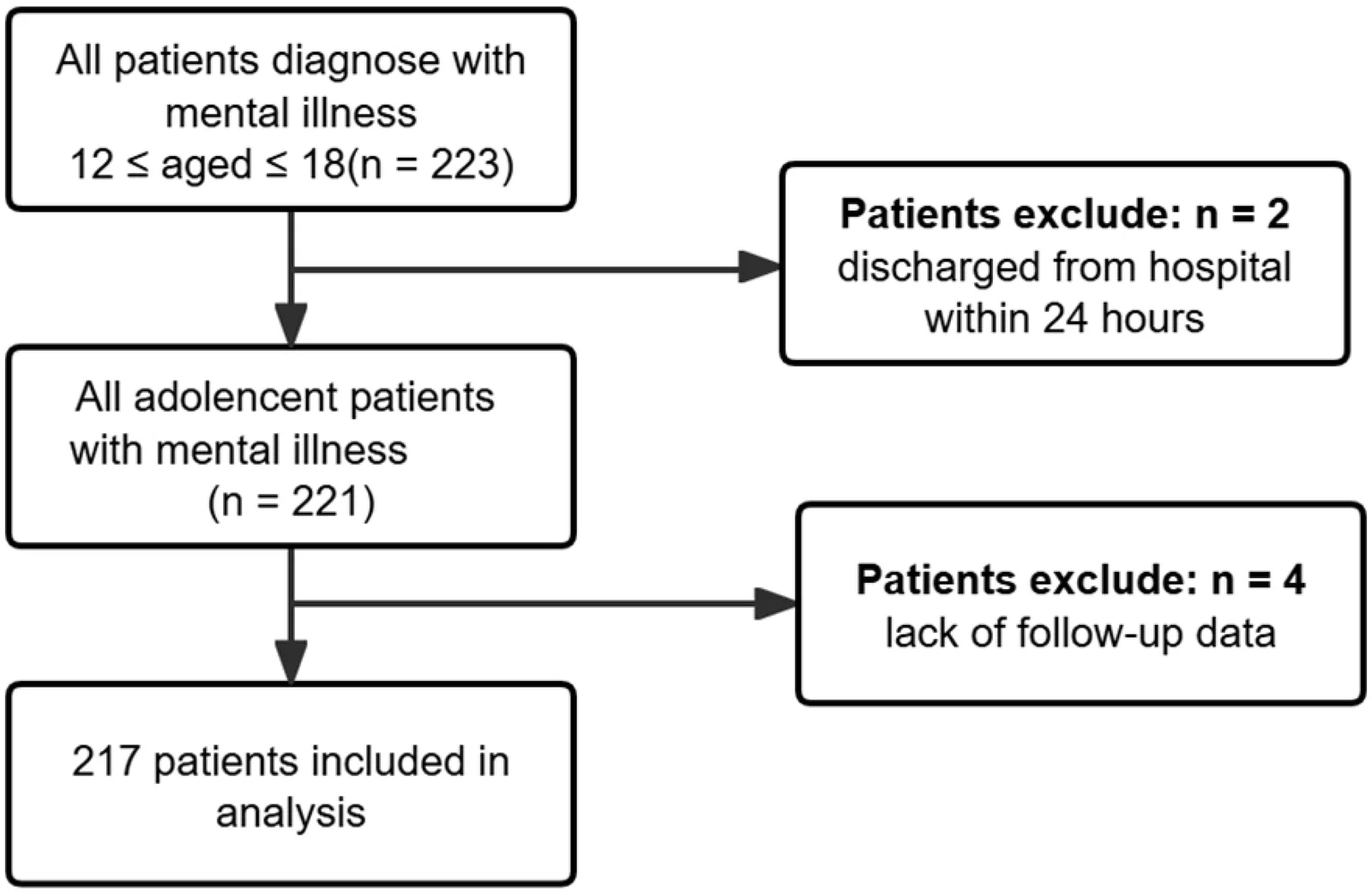
Study flow diagram.

### Social support scale

Social support was assessed using the Social Support Rating Scale (SSRS) originally developed by Xiao Shuiyuan in 1986. The scale is grounded in a tri-dimensional theoretical framework that conceptualizes social support as encompassing three core components: objective support, subjective support, and utilization of support. In this study, we employed the adapted version developed by Ye Yuemei and Dai Xiaoyang in 2008, which was specifically designed for Chinese adolescents and consists of 17 items, α coefficients was 0.81-0.91. Each item is scored on a 5-point Likert scale ranging from 1 to 5. The total score ranges from 17 to 85, with higher scores indicating higher levels of social support. Based on the total score distribution in our sample, participants were categorized into three groups: low social support (scores 17–34), medium social support (scores 35–51), and high social support (scores 52–85).

### Statistical analysis

Patient characteristics were calculated according to the prognosis of the participants. Data were expressed when normally distributed or median and interquartile range (IQR) when skewed. A chi-square test for categorical variables, ANOVA for normally distributed continuous variables, or a Kruskal Wallis test for skewed distributed continuous variables were performed to determine patient variances. Logistic regression models were used to determine the odds ratios (OR) and 95 percent confidence intervals (95% CIs) for the relationship between social support and prognosis. Model 1 was adjusted for adjusted for age, gender, and education. Model 2 was adjusted for Model 1 plus single child status, parental marriage status, ACEs history, NSSI history, suicide idea or attempt, family mental illness history, somatic disease history, diagnosis, the onset of disease, disease course, and pre-hospitalization school absenteeism status. All the analyses were performed with the statistical software packages R 3.3.2 (http://www.R-project.org, The R Foundation) and Free Statistics software version 1.7. A two-tailed test was performed and p < 0.05 was considered statistically significant.

## Results

### Baseline characteristics of the study population

A total of 217 adolescents with mental illness aged 12–18 years were enrolled in this study, including 163 (75.1%) in the school-attending group and 54 (24.9%) in the school-suspended group. The baseline characteristics of the two groups are presented in detail in **Table 1**. There were no significant differences between the two groups in terms of age, gender, only child status, parental education level, history of adverse childhood experiences (ACEs), non-suicidal self-injury (NSSI), suicidal thoughts or behaviors, family history of mental illness, physical disease history, diagnostic subtype, or initial onset of disease (all p > 0.05).

**Table 1:** The characteristics of participants.

Variables	Total (n = 217)	Attend school (n = 163)	Suspend school (n = 54)	*p*
Age, n (%)				0.808
<15y	187 (86.2)	141 (86.5)	46 (85.2)	
≥15y	30 (13.8)	22 (13.5)	8 (14.8)	
Gender, n (%)				0.118
Male	66 (30.4)	45 (27.6)	21 (38.9)	
Female	151 (69.6)	118 (72.4)	33 (61.1)	
Single child, n (%)				0.783
Yes	108 (49.8)	82 (50.3)	26 (48.1)	
No	109 (50.2)	81 (49.7)	28 (51.9)	
Education, n (%)				0.969
Below high school	109 (50.2)	82 (50.3)	27 (50)	
High school and above	108 (49.8)	81 (49.7)	27 (50)	
Parental marriage status, n (%)				**< 0.001**
Married	156 (71.9)	127 (77.9)	29 (53.7)	
Divorced	61 (28.1)	36 (22.1)	25 (46.3)	
ACEs history, n (%)				0.323
Yes	24 (11.1)	20 (12.3)	4 (7.4)	
No	193 (88.9)	143 (87.7)	50 (92.6)	
NSSI history, n (%)				0.782
Yes	104 (47.9)	79 (48.5)	25 (46.3)	
No	113 (52.1)	84 (51.5)	29 (53.7)	
Suicide idea or attempt, n (%)				0.719
Yes	120 (55.3)	89 (54.6)	31 (57.4)	
No	97 (44.7)	74 (45.4)	23 (42.6)	
Family history of mental illness, n (%)				0.485
Yes	30 (13.8)	21 (12.9)	9 (16.7)	
No	187 (86.2)	142 (87.1)	45 (83.3)	
Somatic disease history, n (%)				0.55
Yes	81 (37.3)	59 (36.2)	22 (40.7)	
No	136 (62.7)	104 (63.8)	32 (59.3)	
Diagnosis, n (%)				0.316
F32.Depression	70 (32.3)	57 (35)	13 (24.1)	
F98.Behavioral and emotional disorders with onset usually occurring in childhood and adolescent	88 (40.6)	62 (38)	26 (48.1)	
F31.Bipolar disorder	28 (12.9)	23 (14.1)	5 (9.3)	
F41.Anxiety	16 (7.4)	10 (6.1)	6 (11.1)	
Others	15 (6.9)	11 (6.7)	4 (7.4)	
Onset of disease, n (%)				0.212
Yes	67 (30.9)	54 (33.1)	13 (24.1)	
No	150 (69.1)	109 (66.9)	41 (75.9)	
**Disease course, Median (IQR)**	12.0 (3.0, 24.0)	12.0 (2.0, 24.0)	12.0 (4.0, 26.0)	0.079
Pre-hospitalization absentee status, n (%)				**0.006**
Attend school	138 (63.6)	112 (68.7)	26 (48.1)	
Suspend school	79 (36.4)	51 (31.3)	28 (51.9)	
Social support score, Mean ± SD	45.5 ± 12.2	45.5 ± 12.2	36.2 ± 10.9	**< 0.001**
Social support score, n (%)				**< 0.001**
0-34	36 (17.1)	13 (8.3)	23 (42.6)	
35-51	100 (47.4)	73 (46.5)	27 (50)	
52-85	75 (35.5)	71 (45.2)	4 (7.4)	

Bold values indicate statistically significant between-group differences (P < 0.05). ACEs, adverse childhood experiences; NSSI, non-suicidal self-injury; IQR, interquartile range; SD, standard deviation.

However, significant differences were observed in parental marital status, pre-hospitalization school status, and social support score between the two groups (all p < 0.05). Compared with the school-attending group, the school-suspended group had a significantly higher proportion of parental divorce (46.3% vs. 22.1%, p < 0.001), a higher rate of pre-hospitalization school absenteeism (51.9% vs. 31.3%, p = 0.006), and a significantly lower social support score (36.2 ± 10.9 vs. 45.5 ± 12.2, p < 0.001). The median (interquartile range, IQR) disease course was 12.0 (3.0, 24.0) months in the total population, with no significant difference between the two groups (p = 0.079), but it approached statistical significance.

### Univariate analysis of factors associated with school absenteeism

Univariate logistic regression analysis was performed to explore the crude association between each variable and school absenteeism risk (**Table 2**). The results showed that parental marital status (divorced vs. married), pre-hospitalization school status (suspended vs. attending), social support score (continuous and categorical), and disease course were potentially associated with school absenteeism (all p < 0.05).

**Table 2:** The association of covariates and the prognosis.

Variable	OR_95CI	*P* value
Age (≥15y vs <15y)	1.11 (0.46~2.67)	0.808
Gender (Female vs Male)	0.6 (0.31~1.14)	0.12
Single child status (No vs Yes)	1.09 (0.59~2.02)	0.783
Education (High school and above vs Below high school)	1.01 (0.55~1.87)	0.969
Parental marriage (Divorced vs Married)	3.04 (1.59~5.83)	0.001
ACEs history (No vs Yes)	1.75 (0.57~5.36)	0.329
NSSI history (No vs Yes)	1.09 (0.59~2.02)	0.782
Suicide ideas or attempt (No vs Yes)	0.89 (0.48~1.66)	0.719
Family history of mental illness (No vs Yes)	0.74 (0.32~1.73)	0.486
Somatic disease history (No vs Yes)	0.83 (0.44~1.55)	0.55
Diagnosis (F98.Behavioral and emotional disorders with onset usually occurring in childhood and adolescence vs F32.Depression)	1.84 (0.86~3.92)	0.115
Diagnosis (F31.Bipolar disorder vs F32.Depression)	0.95 (0.31~2.98)	0.934
Diagnosis (F41.Anxiety vs F32.Depression)	2.63 (0.81~8.54)	0.107
Diagnosis (Others vs F32.Depression)	1.59 (0.44~5.81)	0.48
Onset of disease (No vs Yes)	1.56 (0.77~3.16)	0.214
Disease course	1.02 (1~1.04)	0.022
Pre-hospitalization absentee status (Suspend vs Attend)	2.37 (1.26~4.43)	0.007
Social support score (35–51 vs 0-34)	0.21 (0.09~0.47)	<0.001
Social support score (52–85 vs 0-34)	0.03 (0.01~0.11)	<0.001

Specifically, adolescents with divorced parents had a higher risk of school absenteeism than those with married parents (crude OR = 3.04, 95%CI = 1.59–5.83, p = 0.001). Pre-hospitalization school absenteeism was associated with an increased risk of subsequent school absenteeism (crude OR = 2.37, 95%CI = 1.26–4.43, p = 0.007). For social support, each 1-point increase in the continuous score was associated with a 10% reduction in school absenteeism risk (crude OR = 0.90, 95%CI = 0.87–0.94, p < 0.001). Compared with the low social support group (score 17–34), the medium (35–51) and high (52–85) social support groups had significantly lower odds of school absenteeism (crude OR = 0.21, 95%CI = 0.09–0.47, p < 0.001; crude OR = 0.03, 95%CI = 0.01–0.11, p < 0.001, respectively). A longer disease course was also associated with a slightly increased risk of school absenteeism (crude OR = 1.02, 95%CI = 1.00–1.04, p = 0.022). No significant associations were found between other variables (age, gender, only child status, etc.) and school absenteeism (all p > 0.05).

### Multivariate logistic regression analysis of independent predictors of school absenteeism

To identify independent predictors of school absenteeism, three multivariate logistic regression models were constructed (**Table 3**). Model 1 was adjusted for age, gender, and education. Model 2 was adjusted for age, gender, education, single child status, parental marriage status, ACEs history, NSSI history, suicide idea or attempt, family mental illness history, somatic disease history, diagnosis, the initial onset of disease, disease course, pre-hospitalization school absenteeism.

**Table 3:** Association between prognosis and social support score in multivariable logistic regression analyses model.

Social support	n total	Crude model		Mode 1		Model 2	
		OR (95%CI)	*p*	OR (95%CI)	*p*	OR (95%CI)	*p*
social support score	211	0.9 (0.87~0.94)	< 0.001	0.9 (0.87~0.94)	< 0.001	0.91 (0.87~0.96)	< 0.001
17-34 (Low score)	36	1(Ref)		1(Ref)		1(Ref)	
35-51 (Medium score)	100	0.21 (0.09~0.47)	< 0.001	0.21 (0.09~0.47)	< 0.001	0.24 (0.08~0.74)	0.013
52-85 (High score)	75	0.03 (0.01~0.11)	< 0.001	0.03 (0.01~0.11)	< 0.001	0.05 (0.01~0.24)	< 0.001
*p* for trend	211	0.18 (0.1~0.33)	< 0.001	0.18 (0.1~0.33)	< 0.001	0.22 (0.1~0.49)	< 0.001

Crude model: No other covariates were adjusted.

Model 1: adjusted for age, gender, education.

Model 2: adjusted for Model 1 plus single child status, parental marriage status, ACEs history, NSSI history, suicide ideas or attempt, family mental illness history, somatic disease history, diagnosis, the onset of disease, disease course, pre-hospitalization school absentee status.

OR, Odds ratio; CI, Confidence interval.

The results showed that the association between social support and school absenteeism remained stable across all three models. In all three models, each 1-point increase in the social support score was significantly associated with a reduced risk of school absenteeism in adolescents. In the further adjusted Model 2, higher social support was still a strong independent protective factor against school absenteeism: each 1-point increase in the continuous social support score was associated with a 9% reduction in school absenteeism risk (OR = 0.91, 95%CI = 0.87–0.96, p < 0.001). Compared with the low social support group, the medium and high social support groups had significantly lower odds of school absenteeism (OR = 0.24, 95%CI = 0.08–0.74, p = 0.013; OR = 0.05, 95%CI = 0.01–0.24, p < 0.001, respectively).

In addition, the results of trend test showed that p for trend was <0.001 in all three models, clearly confirming a significant dose-response relationship between social support level and the risk of school absenteeism.

### Restricted cubic spline analysis of the dose-response relationship between social support and school absenteeism

Solid and dashed lines indicate predicted values and 95% confidence intervals. They were adjusted for age, gender, education, single child status, parental marriage status, ACEs history, NSSI history, suicide idea or attempt, family mental illness history, somatic disease history, diagnosis, the onset of disease, disease course, pre-hospitalization school absenteeism status. Restricted cubic spline analysis was performed to explore the potential non-linear relationship between social support score and school absenteeism risk, with 44 points as the reference value (**Figure 2**). The results showed that the association between social support score and school absenteeism risk was approximately linear (P for non-linearity = 0.189), indicating that the higher the social support level, the lower the risk of school absenteeism.

**Figure 2:**
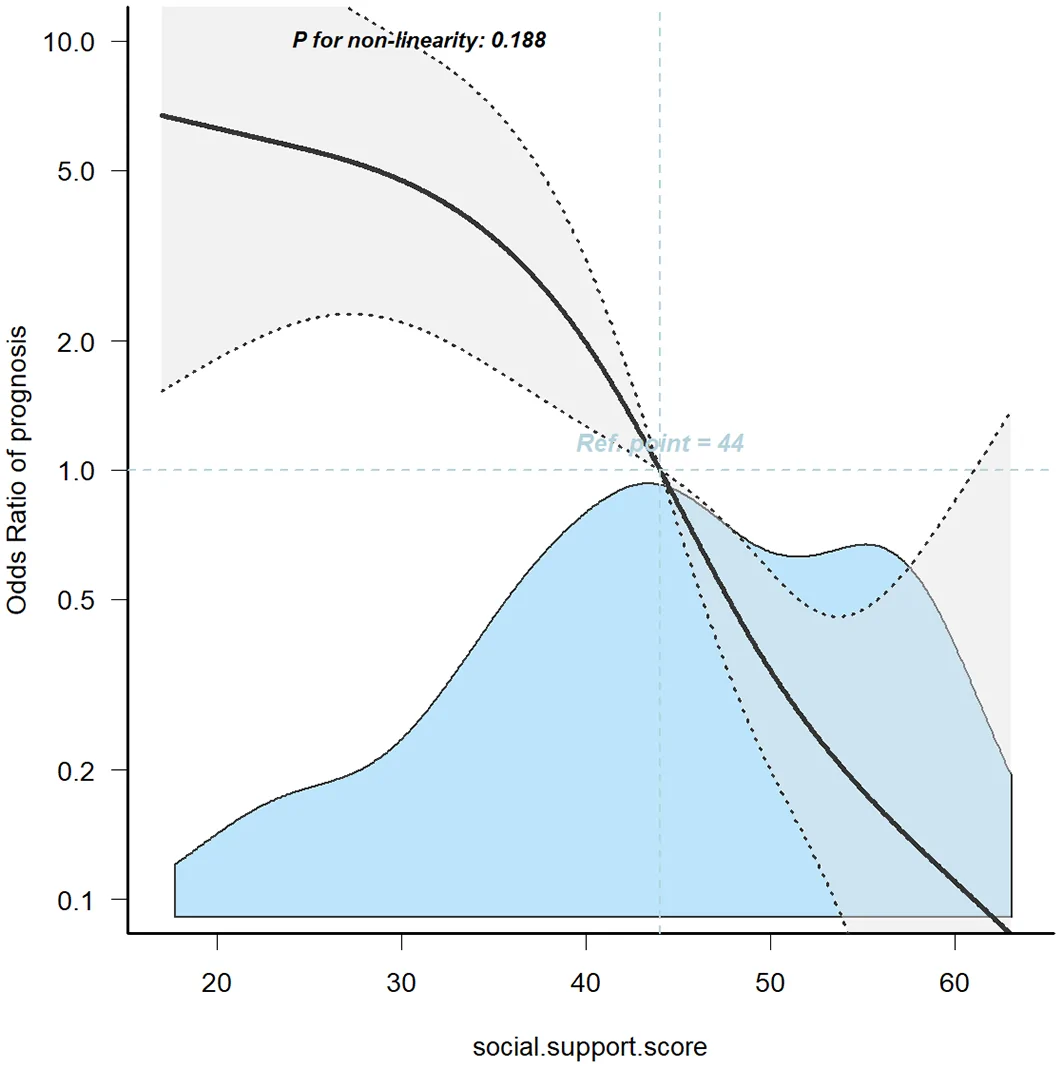
Dose response association between social support score and odds of adverse prognosis. The black solid line depicts the smoothed odds ratio curve; the grey shaded area represents the 95% confidence interval. The reference point was set at a social support score of 44. P for non linearity = 0.189. The blue histogram at the bottom illustrates the distribution of social support scores among participants.

### Sensitivity analysis

Sensitivity analysis was conducted by excluding outliers (social support score < 20 or > 75, n = 8) and re-performing the multivariate logistic regression. The results showed that the OR values and significance of the independent predictors (parental divorce, pre-hospitalization school absenteeism, disease course, and social support) did not change significantly (all p < 0.05), indicating that the study results were robust.

## Discussion

This study adopted a retrospective cohort design and systematically investigated factors associated with school absenteeism among adolescents with mental disorders. The results identified parental divorce, school absenteeism prior to hospital admission, and longer disease course as independent risk factors for school absenteeism, while higher social support served as a protective factor. These findings provide precise entry points for functional rehabilitation and intervention targeting school absenteeism in this population.

Multivariate analysis in this study confirmed that parental divorce was an independent risk factor for school absenteeism in adolescents with mental disorders (OR = 3.04, 95%CI: 1.59–5.83, P = 0.001). The proportion of parental divorce in the school absenteeism group (46.3%) was significantly higher than that in the in-school group (22.1%). Adolescents from stable marital families exhibited significantly better prognostic outcomes compared to those from divorced families. Huang’s study reached similar conclusions, suggesting that family social functioning plays a crucial role in maintaining adolescent emotional health. Adolescents benefit from communication with parents, family support, and parental supervision (20). Changes in family environment (such as divorce or relocation) or health issues of family members can significantly impact adolescents’ psychological state. Individuals affected by parental divorce are at higher risk for various mental health conditions (21), this is consistent with our findings. The psychological distress of parental divorce can persist even into adulthood (11), adults who had poor relationships with their parents during childhood are at a relatively higher risk of experiencing depressive symptoms in later life (22). A complete family structure and a positive family environment can effectively alleviate negative emotions among adolescents with mental disorders, enhance their treatment confidence and social adaptation, and thereby reduce the risk of school absenteeism.

Multivariate analysis confirmed that parental divorce was significantly associated with an elevated risk of school absenteeism among adolescents with mental disorders (OR = 3.04, 95%CI: 1.59–5.83, P = 0.001). The proportion of divorce in the absenteeism group (46.3%) was markedly higher than that in the in-school group (22.1%). Further analysis of divorce duration revealed a cumulative effect: adolescents whose parents had been divorced for more than 5 years exhibited a higher risk of suspension (OR = 3.82, 95%CI: 1.75–8.34) compared to those with a shorter divorce duration (≤3 years, OR = 2.45), suggesting that prolonged exposure to family structural changes may exacerbate maladaptive outcomes. Rather than a direct causal agent, parental divorce should be viewed as a contextual risk factor that disrupts family support systems. According to the family stress model, marital dissolution often reduces parental supervision and emotional availability, while increasing intrafamilial conflict and economic strain—factors that collectively heighten adolescents’ internalizing symptoms and undermine their academic engagement (20). Moreover, the loss of routine and security following divorce may impair adolescents’ emotion regulation capacity, making them more vulnerable to school avoidance behaviors (21). These findings indicate that in clinical practice, the timing of family structure changes should be routinely assessed, and tailored family-based interventions—such as enhancing non-residential parent involvement and strengthening school liaison—are warranted to mitigate the long-term educational cost of parental separation.

Consistent with previous study, the pre-hospital school attendance status, as an independent risk factor for school absenteeism (OR = 2.37, 95%CI=1.26~4.43, P = 0.007), reflects the continuity and cumulative effect of school absenteeism behavior (23).

In this study, the proportion of adolescents in the dropout group who had already been in a state of school absenteeism before hospitalization (51.9%) was much higher than that in the in-school group (31.3%), suggesting that once adolescents drop out of school due to mental disorders, the risk of subsequent persistent school absenteeism will increase significantly if effective intervention measures are not taken in a timely manner.

This may be because during the period of school absenteeism, adolescents are separated from the school’s learning and social environment, lack regular schedules and peer support, and are prone to negative emotions such as loneliness and inferiority, which further aggravate the symptoms of mental disorders, forming a vicious cycle of “school absenteeism - worsening condition - difficulty returning to school”.

The longer students stay away from the school environment, the harder it becomes for them to reintegrate into school life (24). The main reasons include long-term absence leading to a decline in social skills (25). Even if the condition is controlled, it is difficult for them to quickly adapt to campus life, thereby extending the duration of school absenteeism.

Therefore, for adolescents with mental disorders who have shown a tendency to drop out of school or have dropped out of school for the first time, timely implementation of school reentry intervention to help them gradually return to campus is the key to reducing the risk of long-term school dropout (26).

Adolescent mental health is closely related to social support. As a strong protective factor, the linear negative correlation between the level of social support and the risk of school absenteeism (p for non-linearity = 0.189), along with the results of multi-model verification, further highlights the important role and reliability of the social support system in the rehabilitation and school absenteeism of adolescents. Studies have shown that support from family, friends, and other sources significantly improves adolescents’ life satisfaction and reduces depressive symptoms (27). Family support is particularly important for adolescents seeking mental health services, especially when dealing with depression and stress. Peer support is also crucial during adolescence, as it not only enhances psychological well-being but also alleviates loneliness and social anxiety (28). Another study indicates that poor family relationships and insufficient social support are major predictors of anxiety, depression, and post-traumatic stress disorder (PTSD) symptoms in adolescents during the pandemic (29). Social support has a significant protective effect on adolescent mental health, and a lack of support can lead to a range of mental health problems. Promoting support from family, friends, and schools is crucial for improving adolescent mental health (30).

Consistent with the stress-buffering model, social support emerged as a robust protective factor against school absenteeism. In our study, the level of perceived social support (measured by the Multidimensional Scale of Perceived Social Support) showed a significant linear negative correlation with absenteeism risk (p for non-linearity = 0.189), with the protective effect remaining stable across multiple adjustment models. Mechanistically, this protective role operates through three complementary pathways. First, family support—particularly parental involvement in academic monitoring and treatment adherence—directly facilitates structured daily routines, which are critical for adolescents struggling with psychiatric symptoms to maintain school connection (27). Second, peer support serves as a key emotional buffer during adolescence; positive peer interactions alleviate social anxiety and loneliness, which are common barriers to school re-entry following an absence episode (28). Third, school-based support from teachers and counselors fosters a sense of belonging and academic self-efficacy, potentially counteracting the avoidance cycle triggered by academic pressure. Notably, the absence of these support dimensions may leave adolescents with mental disorders isolated from both therapeutic and educational resources, thereby increasing their susceptibility to prolonged suspension. Therefore, multi-tiered interventions that concurrently engage families, classmates, and school staff appear essential for improving re-entry outcomes in this vulnerable population.

It is worth noting that in this study, factors such as age, gender, and diagnostic subtype showed no significant association with the risk of school absenteeism among adolescents, which is inconsistent with the conclusions of some existing studies. The possible reasons are as follows: first, all the subjects included in this study were adolescents aged 12–18 years old, with a small age span and mainly adolescents under 15 years old, so the impact brought by age differences was relatively limited. The 2023 China National Depression Blue Book provides a detailed analysis of adolescent depression symptoms, revealing a significantly high prevalence of depression in this age group. The younger onset of mental disorders has become a pressing issue in the global public health domain (1, 2). Studies indicate a significant increase in the incidence of mental disorders such as depression (3), anxiety, and bipolar disorder (4) among adolescents and young adults, primarily due to the rapid changes and pressures of modern society (31). This trend may be attributed to excessive use of social media (32), increased academic and occupational pressures, changes in family structure, and weakened social support systems; second, in terms of gender, the proportion of females in both groups was higher than that of males, which may be related to the fact that females are more prone to emotional mental disorders. This result is consistent with previous studies that the incidence of adolescent mental illness is twice as high in girls as in boys (33) consistent results with other studies (34, 35), which indicated females have a higher incidence of mental illness in early adolescence. This phenomenon is likely linked to the interaction of biological, psychological, and social factors. Hormonal fluctuations during adolescence, such as changes in estrogen and progesterone levels, can affect neural pathways related to emotion regulation (36), increasing the risk of mental disorders. Social and cultural expectations regarding gender roles, appearance standards, and peer comparisons further exacerbate psychological stress (37). but there is no significant difference in the severity of the disease between females and males; third, no difference was found in diagnostic subtypes, which may be because the impact of different types of mental disorders on adolescents’ school attendance is mainly achieved through mediating factors such as disease severity and emotional regulation ability, rather than the diagnostic type itself.

Several demographic and clinical variables, including age, gender, and diagnostic subtype, did not reach statistical significance in predicting absenteeism. This may be attributable to the restricted age range (12–18 years, with a predominance of participants under 15) and the relatively balanced gender distribution in terms of illness severity, which likely limited the detectability of age- or sex-specific effects. More importantly, the absence of a diagnostic subtype effect suggests that the risk of school absenteeism may be driven more by transdiagnostic factors—such as emotion dysregulation and functional impairment—rather than by diagnostic labels per se. This aligns with the growing consensus that dimensional assessments of psychosocial functioning could yield more clinically actionable information than categorical diagnoses in predicting real-world outcomes. In contrast, a longer disease course was identified as a significant independent risk factor (OR = 1.02, 95%CI = 1.00–1.04, p = 0.022). Prolonged illness duration may erode adolescents’ academic self-concept and social networks over time, increasing the perceived difficulty of re-integrating into school. This cumulative disadvantage underscores the importance of early intervention not only for symptom remission but also for preserving educational continuity from the onset of psychiatric illness.

### Limitations

This study still has limitations. First, this study is a retrospective cohort study which cannot clarify the causal relationship between various factors and school absenteeism. Prospective studies can be conducted in the future. Second, the study sample was derived from a single research center, which may lead to selection bias, and further verification is needed through multicenter and large-sample studies. Finally, this study did not analyze in detail the specific associations between different dimensions of social support (e.g., family support, peer support) and school absenteeism. Further detailed research can be carried out to provide more precise evidence for targeted interventions.

Several limitations should be considered when interpreting our findings. First, although we identified significant associations between parental divorce, social support, and school absenteeism, the cross-sectional nature of the study precludes causal inference. Moreover, we did not collect detailed data on key confounders such as family socioeconomic status, parental educational attainment, or school-level academic pressure—factors that may influence both family stability and educational outcomes. Their absence raises the possibility of residual confounding, particularly in the observed divorce-absenteeism relationship. Second, while we retrieved disease course information from medical records, these data were retrospectively extracted and may be subject to recall or documentation inaccuracies, especially for onset time and prior absenteeism episodes. The relatively short observation window (i.e., hospitalization period) also limits our ability to capture dynamic changes in school attendance post-discharge. Third, the single-center design and the relatively modest sample size restricted our capacity to conduct stratified analyses by social support subdimensions (family vs. peer vs. school) or by specific diagnostic categories, which might have masked heterogeneous effects across subgroups. Finally, all participants were recruited from a psychiatric inpatient setting in a specific region, which may limit the generalizability of our findings to outpatient or community-based adolescent populations with milder symptom severity. Future multi-center prospective cohorts with comprehensive covariate adjustment and longer follow-up are warranted to validate and extend these observations.

## Conclusion

Our study found that parental divorce, school absenteeism prior to hospitalization, and a longer course of disease are independent risk factors for school absenteeism among adolescents with mental disorders, while a higher level of social support is a strong protective factor. These findings suggest that targeted interventions should be developed in clinical practice, with a focus on maintaining family stability, improving social support, and providing early intervention for adolescents with school absenteeism tendencies, so as to reduce the risk of school absenteeism and promote functional recovery in this vulnerable group.

## Data Availability

The raw data supporting the conclusions of this article will be made available by the authors, without undue reservation.
